# Cancer Stem Cells in Glioblastoma Multiforme

**DOI:** 10.3389/fsurg.2016.00048

**Published:** 2016-08-26

**Authors:** Amy Bradshaw, Agadha Wickremesekera, Helen D. Brasch, Alice M. Chibnall, Paul F. Davis, Swee T. Tan, Tinte Itinteang

**Affiliations:** ^1^Gillies McIndoe Research Institute, Wellington, New Zealand; ^2^Department of Neurosurgery, Wellington Regional Hospital, Wellington, New Zealand; ^3^Wellington Regional Plastic, Maxillofacial and Burns Unit, Hutt Hospital, Wellington, New Zealand

**Keywords:** glioblastoma multiforme, embryonic, cancer, stem cells, expression, hierarchy

## Abstract

**Aim:**

To identify and characterize cancer stem cells (CSC) in glioblastoma multiforme (GBM).

**Methods:**

Four-micrometer thick formalin-fixed paraffin-embedded GBM samples from six patients underwent 3,3-diaminobenzidine (DAB) and immunofluorescent (IF) immunohistochemical (IHC) staining for the embryonic stem cell (ESC) markers NANOG, OCT4, SALL4, SOX2, and pSTAT3. IF IHC staining was performed to demonstrate co-expression of these markers with GFAP. The protein expression and the transcriptional activities of the genes encoding NANOG, OCT4, SOX2, SALL4, and STAT3 were investigated using Western blotting (WB) and NanoString gene expression analysis, respectively.

**Results:**

DAB and IF IHC staining demonstrated the presence of a CSC population expressing NANOG, OCT4, SOX2, SALL4, and pSTAT3 with the almost ubiquitous presence of SOX2 and a relatively low abundance of OCT4, within GBM. The expression of NANOG, SOX2 and, pSTAT3 but, not OCT and SALL4, was confirmed by WB. NanoString gene analysis demonstrated transcriptional activation of NANOG, OCT4, SALL4, STAT3, and SOX2 in GBM.

**Conclusion:**

This study demonstrated a population of CSCs within GBM characterized by the expression of the CSC markers NANOG, SALL4, SOX2, pSTAT3 and OCT4 at the protein and mRNA levels. The almost ubiquitous presence of SOX2 and a relatively low abundance of OCT4 would support the putative existence of a stem cell hierarchy within GBM.

## Introduction

Glioblastoma multiforme (GBM), a grade 4 astrocytoma, is the most aggressive primary brain tumor with a 5-year survival of 2%, despite intensive research (1, 2) and the tumor usually recurs following surgical resection, radiotherapy, and chemotherapy (3, 4). This poor prognosis has been attributed to the initiation, propagation, and differentiation of cancer stem cells (CSCs) (3, 5, 6).

The CSC concept proposes that a cancer originates from a small population of CSCs either by the acquisition of mutations in normal embryonic stem cells (ESCs) or progenitor cells that were imbued with the abilities for uncontrolled growth and propagation (7, 8). While the tumorigenic CSCs are proposed to be the driving force behind tumor growth, the bulk of the tumor consists of non-tumorigenic cancer cells that have differentiated from these CSCs, leading to a vast cellular heterogeneity (8, 9). Furthermore, when exposed to certain epigenetic or environmental factors, the downstream cancer cells can be reprogramed to acquire stem cell properties (10).

Embryonic stem cells, which were originally isolated from cells of the inner cell mass of an early mammalian embryo (blastocyst) (11), possess the ability for perpetual propagation and differentiation into all cell lineages (12). ESCs and their downstream progenitors express a variety of characteristic proteins, including cell surface markers and transcription factors (13). CSCs in GBM bear the characteristics of ESCs by their expression of similar proteins, making it possible to both characterize and isolate CSCs in GBM (13, 14). A recent report demonstrates an ESC-like signature in high grade (grades 3 and 4) gliomas with upregulation of NANOG, KLF4, OCT4, and SOX2 proteins (15). Upregulation of these proteins has been correlated with poorer survival in both high- and low-grade gliomas (15, 16), although inclusion of gliomas of different grades and their analysis as a single entity means that this study inherently lacks the specificity needed for the characterization of a unique CSC population in GBM. A recent review points to CSCs in GBM possessing a hierarchy, with overlapping phenotypes expressing upstream (ESC) and downstream (progenitor cell) markers (17).

While many CSC markers have been associated with CSCs, there is evidence indicating that some proteins play a greater role than others in maintaining ESC capabilities. NANOG, OCT4, and SOX2 are transcription factors that have been proposed to function synergistically to maintain ESC pluripotency and self-renewal (18). Expression of this protein trio has also been linked to aggressiveness of GBM, and all three are expressed in most gliomas (19). SALL4 is another transcription factor responsible for zygotic survival and ESC pluripotency (20, 21). Additionally, SALL4 physically interacts with NANOG and is associated with OCT4 and SOX2 (22, 23). pSTAT3, a signaling and transcription activating molecule, is also involved in ESC pluripotency (24) and is proposed to engender expression of other ESC-associated proteins such as SOX2 and SALL4 (25).

This study aimed to identify and characterize the CSC population within GBM, using the ESC markers SOX2, OCT4, pSTAT3, SALL4, and NANOG at both the transcriptional and translational levels.

## Materials and Methods

### Tissue Samples

Six GBM tissue samples from three male and three female patients aged 42–81 (mean, 64.2) years were sourced from the Gillies McIndoe Research Institute Tissue Bank, and used in a study approved by the Central Health and Disabilities Ethics Committee (ref. no. 15CEN28).

### Histochemical and Immunohistochemical Staining

Four-micrometer thick formalin-fixed paraffin-embedded sections of GBM from six patients were used for hematoxylin and eosin staining to confirm the diagnosis of GBM by an anatomical pathologist (HDB). 3,3-Diaminobenzidine (DAB) and immunofluorescent (IF) immunohistochemical (IHC) staining of these sections was then performed using the Leica Bond Rx auto-stainer (Leica, Nussloch, Germany) as previously described (26). DAB IHC staining for GFAP (cat# PA0026, Leica), NANOG (1:100; cat# ab80892, Abcam, Cambridge, UK), SOX2 (1:500; cat# PA-094, Thermo Fisher Scientific, Rockford, IL, USA), SALL4 (1:30; cat# CM385M-16, Cell Marque, Rocklin, CA, USA), pSTAT3 (1:100; cat# 9145, Cell Signaling Technology, Danvers, MA, USA), and OCT4 (1:1000; cat# ab109183, Abcam) diluted with Bond™ primary antibody diluent (cat# AR9352, Leica) was undertaken for all GBM tissue samples. IF IHC staining was performed on two representative GBM tissue samples from the original cohort of patients included in DAB IHC staining, using identical primary antibodies and concentrations with an appropriate fluorescent secondary antibody. All IF IHC-stained slides were mounted using Vectashield HardSet anti-fade mounting medium with 4,6-diamidino-2-phenylindole (cat# H-1500, Vector Laboratories, Burlingame, CA, USA).

Positive control human tissues for the primary antibodies were seminoma for NANOG, SALL4, and OCT4, skin for SOX2, and tonsil for pSTAT3. A secondary and tertiary only negative control staining by omitting the primary antibodies was performed on a GBM sample randomly selected from the original cohort of GBM samples used for DAB IHC staining (Figure S1 in Supplementary Material).

### Image Analysis

All DAB IHC-stained slides were visualized with an Olympus BX53 light microscope (Tokyo, Japan) and the images were captured with the CellSens 2.0 software (Olympus). IF IHC stained-slides were viewed and the images were captured using an Olympus FV1200 biological confocal laser-scanning microscope and processed with cellSens Dimension 1.11 software using 2D deconvolution algorithm (Olympus).

### Western Blotting

Five snap-frozen GBM samples, from the original cohort of six patients included in DAB IHC staining, were washed in 1× PBS and homogenized in RIPA buffer (cat# R0278, Sigma-Aldrich, St Lewis, MA, USA) supplemented with Halt™ Protease and Phosphatase Inhibitor Cocktail (cat# 1861281, Thermo Scientific, Waltham, MA, USA) and dithiothreitol (DTT) (cat# DTT-RO, Sigma-Aldrich). Protein was precipitated using a Calbiochem^®^ ProteoExtract^®^ Protein Precipitation Kit (cat# 539180, EMD Millipore Corp, Billerice, MA, USA) for 1 h at −20°C, washed and re-suspended in 1× Laemmli sample buffer (cat# 161-0737, Bio-Rad, Hercules, CA, USA) with 1% DTT. Equal amounts of protein were heated at 85°C and separated on Bolt™ 4–12% Bis–Tris Plus gels (cat# NW04120BOX, Invitrogen, Carlsbad, CA, USA) via electrophoresis. Separated protein was transferred to a nitrocellulose membrane (cat# IB23001, Life Technologies, Carlsbad, CA, USA) and blocked in 1× tris-buffered saline (pH 7.4) containing 0.1% Tween-20 (TBST) containing 2% skim milk powder for 90 min at 4°C. Primary antibody probing for each CSC marker was overnight in TBST at 4°C with the following primary antibodies at the given concentrations: rabbit monoclonal anti-OCT4 (1:1000; cat# ab109183, Abcam), anti-pSTAT3 (1:2000; cat# ab9145, Abcam), anti-SOX2 (1:5000; cat# PA1-094, Thermo Fisher, Scoresby, VIC, Australia), and anti-NANOG (1:2000; cat# ab47102, Abcam). Secondary antibody probing was in 1× TBST for 60 min at 4°C with goat anti-rabbit horseradish peroxidase (HRP; 1:10,000; cat# A16110, Thermo Fisher). Beta-actin antibody probing was performed with the iBind™ Flex device (cat# SLF2000, Life Technologies) using primary mouse monoclonal anti-β-actin (1:2000 cat# ab8226, Abcam) and secondary donkey anti-mouse Alexa fluor 488 (1:2000 cat# A21202, Thermo Fisher). Clarity Western ECL (cat# 1705061, Bio-Rad) was used as the substrate for visualizing HRP-detected protein bands, and the Chemi Doc MP Imaging System (Bio-Rad) and Image Lab 5.0 software (Bio-Rad) were used for both HRP and fluorescent band detection and analysis.

Positive controls for the markers examined were NTERA2 for OCT4; NTERA2 and human placenta for SOX2; mouse lung and human liver for pSTAT3; 3T3 cell lysate for NANOG. Negative controls were HeLa for OCT4; human placenta for pSTAT3; SY5Y for NANOG. No negative tissues or lysates could be found for SOX2.

### NanoString Gene Expression Analysis

Total RNA was extracted from ~20 mg of six snap-frozen GBM tissue from the same cohort of patients included in DAB IHC analysis, using the MagJET RNA kit (cat# k2731, Thermo Scientific) and the Kingfisher Duo RNA extraction machine (Thermo Scientific). All samples were quantitated and quality controlled with the NanoDrop 2000 Spectrophotometer (Thermo Scientific) and the Qubit 2.0 Fluormeter (Thermo Scientific). The samples with A260/A230 ≥1.5 and A260/A280 ~2 were used for further analysis. The integrity of the RNA was assessed by New Zealand Genomics Ltd. (Dunedin, NZ) using Agilent 2100 BioAnalyzer (Agilent Technologies, Santa Clara, CA, USA). The isolated RNA was then subjected to NanoString nCounter™ Gene Expression Assay (NanoString Technologies, Seattle, WA, USA) as completed by New Zealand Genomics Ltd. (Dunedin, New Zealand), according to the manufacturer’s protocol. Probes for the genes encoding NANOG (XM_011520850.1), SOX2 (NM_003106.3), SALL4 (NM_020436.3), OCT4 (NM_001159542.1), and STAT3 (NM_139276.2) and the housekeeping gene GAPDH (NM_002046.3) were designed and synthesized by NanoString Technologies. Raw data were analyzed using nSolver™ software (NanoString Technologies) using standard settings and was normalized against the housekeeping gene.

## Results

### Histochemical and 3,3-Diaminobenzidine Immunohistochemical Staining

**Figure 1:**
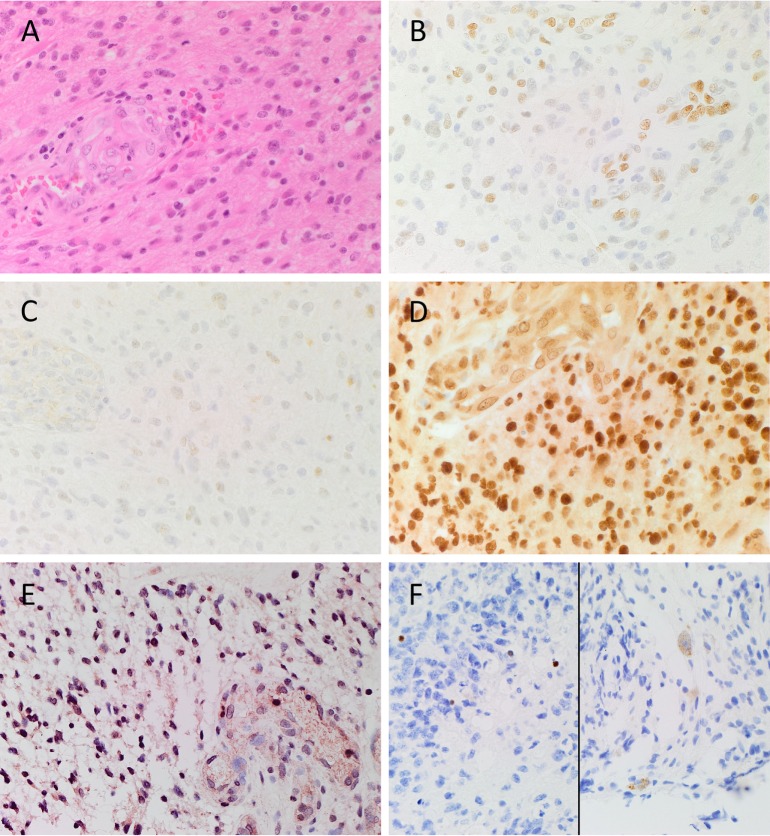
**Representative image of H&E stained slides showing the presence of GBM (A) and DAB IHC-stained slides of GBM tissue demonstrating expression of CSC markers (B–F)**. pSTAT3 [**(B)**, brown] was expressed in the nuclei of tumor and endothelium of the microvessels throughout the sample. SALL4 [**(C)**, brown] was predominantly expressed on the nuclei of the tumor cells, and the cytoplasm of the endothelial cells lining the microvessels. SOX2 [**(D)**, brown] displayed strong nuclear staining of the tumor cells, and moderate cytoplasmic staining of the endothelium of the microvessels. Nuclear expression of NANOG [**(E)**, pink/red] was observed on the tumor cells, and to the lesser extent on the endothelium of the microvessels. Nuclear and cytoplasmic staining of OCT4 [**(F)**, brown] was observed in few tumor cells. Cell nuclei were counterstained with hematoxylin [**(A–F)**, blue]. Original magnification: 400X.

Hematoxylin and eosin stain (Figure 1A) confirmed the diagnosis of grade 4 astrocytoma on all 6 GBM samples. Positive staining for pSTAT3 (Figure 1B, brown), SALL4 (Figure 1C, brown), and SOX2 (Figure 1D, brown) was observed in tumor cells and within areas of endothelial proliferation. pSTAT3 showed consistent nuclear staining throughout the sample (Figure 1B, brown), and SALL4 staining was localized predominantly to the nuclei of tumor cells but was mostly cytoplasmic in the proliferative endothelium (Figure 1C, brown). SOX2 staining showed strong nuclear staining in tumor cells that lessened in intensity within areas of endothelial proliferation, with a consistent, moderate level of cytoplasmic staining throughout the entire sample (Figure 1D, brown). NANOG was localized to the nuclei of tumor cells but was not present in the endothelium (Figure 1E, pink). OCT4 staining was scarce in all samples but showed differential staining patterns, with some cells exhibiting nuclear (Figure 1F, brown, *left*) and others cytoplasmic (Figure 1F, brown, *right*) staining.

Expected staining patterns for pSTAT3 (Figure S1A in Supplementary Material, brown), SALL4 (Figure S1B in Supplementary Material, brown), SOX2 (Figure S1C in Supplementary Material, brown), NANOG (Figure S1D in Supplementary Material, brown), and OCT4 (Figure S1E in Supplementary Material, brown) were demonstrated in the respective positive controls. Staining with the omission of the primary antibodies in a GBM sample provided an appropriate negative control (Figure S1F in Supplementary Material).

### Immunofluorescent Immunohistochemical Staining

**Figure 2:**
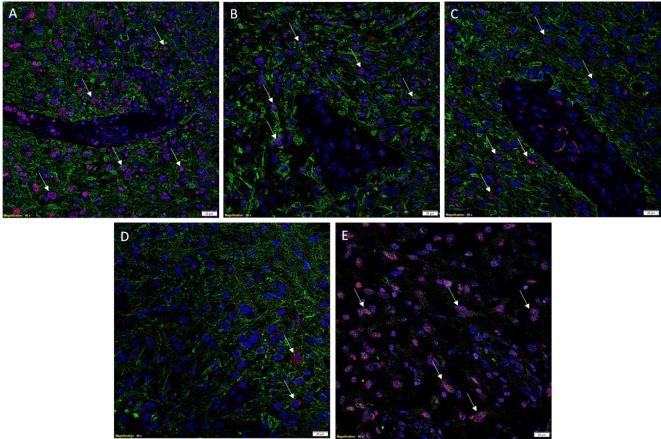
**Representative images of IF IHC-stained sections of GBM tissue for ESC markers**. SOX2 [**(A)**, red, *arrows*], NANOG [**(B)**, red, *arrows*], and pSTAT3 [**(C)**, red, *arrows*] all showed nuclear expression on GFAP+ tumor cells [**(A–C)**, green]. OCT4 [**(D)**, red, *arrows*] staining was scarce and solely cytoplasmic in GFAP+ tumor cells. SALL4 [**(E)**, green] and SOX2 [**(E)**, red] were co-expressed (*arrows*) in the nuclei of some tumor cells, with SALL4 also staining SOX2–negative cells. Cell nuclei were counterstained with 4′, 6′-diamidino-2-phenylindole [(A–E), blue]. Scale bars: 20 μm.

To investigate co-expression of the ESC markers, IF IHC staining was performed on two representative GBM samples used for DAB IHC staining. To identify GBM tumor cells, GFAP (Figures 2A–D, green) was utilized as a marker for glial cells (27, 28). A substantial number of GFAP^+^ cells also expressed SOX2 (Figure 2A, red, *arrows*), NANOG (Figure 2B, red, *arrows*), and pSTAT3 (Figure 2C, red, *arrows*) with relatively low expression of OCT4 (Figure 2D, red, *arrow*). To examine expression of SALL4, we counterstained the same samples for SOX2 (Figure 2E, red) and SALL4 (Figure 2E, green) demonstrating the expression of both markers in the same nuclei (Figure 2E, *arrows*). The vasculature (Figures 2A–C, *arrows*) did not demonstrate expression of GFAP (Figures 2A–C, green), as expected, along with minimal expression of the aforementioned ESC markers. Images of the individual stains are presented in Figure S2 in Supplementary Material. A GBM sample used as a negative control by omitting the primary antibodies, demonstrated the specificity of the antibodies used (data not shown).

### Western Blotting

**Figure 3:**
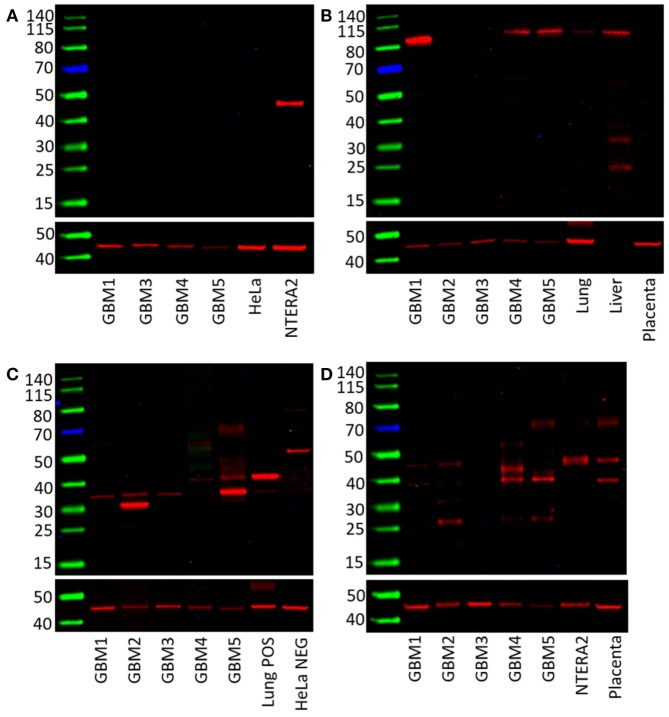
**Western blots of five GBM tissue samples**. OCT4 was not detected **(A)**, pSTAT3 at ~90 kDa was found in three out of five samples **(B)**, NANOG was present in all five samples with multiple bands at approximately 40 and 31 kDa **(C)**. SOX2 was detected in four out of five samples with bands at approximately 45 and 38 kDa **(D)**.

The presence of OCT4, NANOG, pSTAT3, and SOX2 in GBM samples was also examined by WB. OCT4 was below the detection level in all four GBM samples compared with NTERA2 cell lysate used as a positive control, which showed a band of approximately 46 kDa (Figure 3A). pSTAT3 was expressed in three out of five GBM samples at ~90 kDa (Figure 3B). NANOG was present in three out of four GBM samples, although multiple bands were detected with the antibody at approximately 40 and 35 kDa (Figure 3C). SOX2 was detected in four out of five samples and multiple bands at approximately 45 and 38 kDa were observed (Figure 3D). WB data for SALL4 has not been included due to antibody difficulties involving non-specific binding (data not shown).

### NanoString Gene Expression Analysis

**Figure 4:**
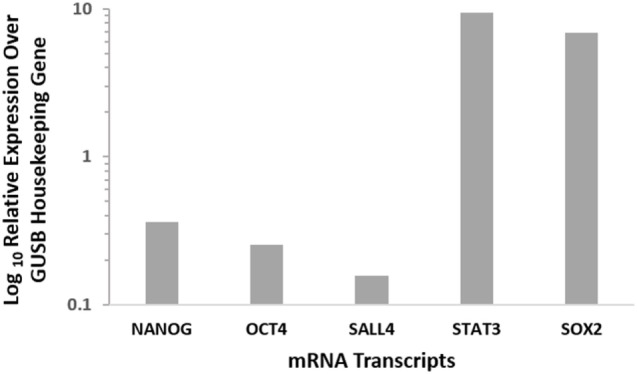
**Relative expression of CSC mRNA transcripts in six GBM samples showing the presence of all 5 markers at varying levels**. NANOG, OCT4, and SALL4 showed relatively low mRNA expression, while STAT3 and SOX2 displayed high levels of mRNA expression. Expression is depicted relative to the housekeeper GUSB.

mRNA quantification was performed for NANOG, OCT4, SALL4, STAT3, and SOX2 to investigate the presence of transcription activation of these markers in GBM. The expression values were normalized to that of the housekeeping gene GUSB and showed that all five markers were expressed in GBM samples (Figure 4).

## Discussion

The CSC concept of cancer proposes that a tumor is generated by a small number of cells that possess the ability for indefinite self-renewal and differentiation into multiple cell types (7, 8). There is growing evidence in support of this concept, with CSCs being identified and characterized in many cancer types (17). The presence of CSCs in brain tumors was first reported by Singh et al. (6) and has been linked to tumor aggression and decreased life expectancy (8, 29). Furthermore, the presence of distinct ESC markers within GBM tumors has also been associated with poor outcomes (30–32). Activation of these protein markers in cancer cells imbues them with ESC characteristics such as indefinite self-renewal and pluripotency, and results in CSCs (17). We have demonstrated the expression of the ESC markers NANOG, SALL4, OCT4, SOX2, and pSTAT3 in GBM using DAB IHC staining (Figures 1A–F), WB (Figures 3A–D) and NanoString analysis (Figure 4). IHC staining showed the expression of all five ESC markers within the GBM samples examined (Figure 1), a finding that was corroborated by NanoString mRNA analysis in all six GBM samples. In this report, we have also demonstrated the expression of pSTAT3, NANOG, and SOX2 using WB analysis. Due to improperly functioning antibodies, the presence of SALL4 could not be determined by WB, and this warrants further investigation. It is intriguing that OCT4 was below detection levels by WB despite being observed by both IHC staining and NanoString analysis.

As NanoString analysis showed relatively low transcript numbers for OCT4 (Figure 4) and DAB IHC staining showed OCT4 was expressed by very few cells within the tumor (Figure 1F), we infer that OCT4 was too low in abundance to be detected by WB. Possible reasons for this include sampling bias and/or relatively low levels of protein within the GBM tissues examined. This may in part be explained by the inherent intra-tumor heterogenity within GBM tumors, with previous studies demonstrating spectral expression patterns for both SOX2 and pSTAT3 including GBM (1, 33, 34). This is further supported by the well recognized inter-tumor heterogenity observed in GBM, which potentially provides each tumor with a unique stem cell signature (10, 17, 35, 36). This may also account for the variation of the number of detected bands for NANOG and SOX2 on WB (Figures 3C,D). Different-sized bands may represent detection of different protein isoforms that have undergone post-translational modification (PTM). Different sized variants of NANOG have been identified previously with native NANOG (NANOG a) at 34.2 kDa, NANOG b at 34.4 kDa, and NANOG c at 31.9 kDa (37). Furthermore, NANOG can undergo phosphorylation at several residues (38) and is also bound by the ubiquitin–proteasome system (39), both of which have the potential to alter band size on a WB. As PTM can alter protein function (39), the observation that three out of the four GBM samples analyzed contained only the 35 kDa variant (possibly corresponding to NANOG c with some PTMs, as the larger 40 kDa band in the positive 3T3 lysate control is likely to be NANOG a/b) indicates that the types of modified protein present within a tumor may be significant and that the presence of NANOG c in GBM may even be a predictor of tumor aggression.

SOX2 can also undergo phosphorylation, SUMOylation, and glycosylation (40, 41). Similar to NANOG, SOX2 PTMs can upregulate or downregulate SOX2 function, thus influencing stem cell function (39, 41). It is therefore conceivable that multiple modified and/or isoforms of particular ESC markers may be present in any given tumor.

This study demonstrates relative abundance of ESC markers OCT4, SOX2, SALL4, NANOG, and pSTAT3. Our data indicates the possibility that different isoforms or modified versions of SOX2 and NANOG may exist within different GBM tumors, and that a particular isoform present may influence tumor growth and tumor aggression in specific ways, although this remains a topic for further study. Additionally, in contrast to previous studies (15, 42), IHC staining and NanoString analysis in our study revealed relatively low expression levels of OCT4 at the transcriptional activation and corresponding protein levels. We speculate that the relatively low number of cells expressing OCT4 represent the most primitive stem cell population within GBM and that they may potentially give rise to the remaining down-stream cells within the GBM tumor. Similarly, the almost ubiquitous abundance of SOX2 within GBM suggests that this marker is expressed on the more differentiated cells reflecting SOX2 as a putative progenitor cell marker within the GBM samples used in this study. Further investigation in this area may lead to the possibility of tailoring future treatment of GBM by targeting the most primitive OCT4 + CSC subpopulation.

Although a limitation of this study is the relatively small sample size, the novel findings we present lay a platform for future studies to better understand the precise role of CSCs in GBM.

### Ethics Approval

Central Health and Disabilities Ethics Committee (ref. no. 15CEN28).

## Author Contributions

TI and ST formulated the study hypothesis. TI, AW, and ST designed the study. TI, HB, AB, AW, PD, and ST interpreted the DAB IHC data. TI, AW, and ST interpreted the IF IHC data. AB performed WB analysis. AB, TI, AW, PD, and ST interpreted the WB data. AC processed the tissues for NanoString analysis and interpreted the data. AB drafted the manuscript. All authors commented on and approved the manuscript.

## Conflict of Interest Statement

The authors declare that the research was conducted in the absence of any commercial or financial relationships that could be construed as a potential conflict of interest. TI, PD, and ST are inventors of the PCT patent application (No. PCT/NZ2015/050108) Cancer Diagnosis and Therapy.

## References

[1] Schmitz M Temme A Senner V Ebner R Schwind S Stevanovic S Identification of SOX2 as a novel glioma-associated antigen and potential target for T cell-based immunotherapy. Br J Cancer (2007) 96(8):1293–301. 10.1038/sj.bjc.6603802 17375044 PMC2360145

[2] Surawicz TS Davis F Freels S Laws ER Menck HR. Brain tumor survival: results from the National Cancer Data Base. J Neurooncol (1998) 40(2):151–60. 10.1023/a:1006091608586 9892097

[3] Frosina G. Frontiers in targeting glioma stem cells. Eur J Cancer (2011) 47(4):496–507. 10.1016/j.ejca.2010.11.017 21185169

[4] Stupp R Hegi ME Mason WP van den Bent MJ Taphoorn MJ Janzer RC Effects of radiotherapy with concomitant and adjuvant temozolomide versus radiotherapy alone on survival in glioblastoma in a randomised phase III study: 5-year analysis of the EORTC-NCIC trial. Lancet Oncol (2009) 10(5):459–66. 10.1016/S1470-2045(09)70025-7 19269895

[5] Frosina G. The bright and the dark sides of DNA repair in stem cells. J Biomed Biotechnol (2010) 2010:845396. 10.1155/2010/845396 20396397 PMC2852612

[6] Singh SK Clarke ID Terasaki M Bonn VE Hawkins C Squire J Identification of a cancer stem cell in human brain tumors. Cancer Res (2003) 63(18):5821–8. 14522905

[7] Rajaraman R The origin of tumor cell heterogeneity. IUP J Biotechnol (2010) 4(4):7–43.

[8] Shipitsin M Polyak K. The cancer stem cell hypothesis: in search of definitions, markers, and relevance. Lab Invest (2008) 88(5):459–63. 10.1038/labinvest.2008.14 18379567 PMC3702270

[9] Adams JM Strasser A. Is tumor growth sustained by rare cancer stem cells or dominant clones? Cancer Res (2008) 68(11):4018–21. 10.1158/0008-5472.CAN-07-6334 18519656

[10] Safa AR Saadatzadeh MR Cohen-Gadol AA Pollok KE Bijangi-Vishehsaraei K Glioblastoma stem cells (GSCs) epigenetic plasticity and interconversion between differentiated non-GSCs and GSCs. Genes Dis (2015) 2(2):152–63. 10.1016/j.gendis.2015.02.001 26137500 PMC4484766

[11] Evans MJ Kaufman MH Establishment in culture of pluripotential cells from mouse embryos. Nature (1981) 292(5819):154–6. 10.1038/292154a0 7242681

[12] Thomson JA Itskovitz-Eldor J Shapiro SS Waknitz MA Swiergiel JJ Marshall VS Embryonic stem cell lines derived from human blastocysts. Science (1998) 282(5391):1145–7. 10.1126/science.282.5391.1145 9804556

[13] Zhao W Ji X Zhang F Li L Ma L. Embryonic stem cell markers. Molecules (2012) 17(6):6196–236. 10.3390/molecules17066196 22634835 PMC6268870

[14] Schoenhals M Kassambara A De Vos J Hose D Moreaux J Klein B. Embryonic stem cell markers expression in cancers. Biochem Biophys Res Commun (2009) 383(2):157–62. 10.1016/j.bbrc.2009.02.156 19268426

[15] Elsir T Edqvist PH Carlson J Ribom D Bergqvist M Ekman S A study of embryonic stem cell-related proteins in human astrocytomas: Identification of Nanog as a predictor of survival. Int J Cancer (2014) 134(5):1123–31. 10.1002/ijc.28441 24037901

[16] Holmberg J He X Peredo I Orrego A Hesselager G Ericsson C Activation of neural and pluripotent stem cell signatures correlates with increased malignancy in human glioma. PLoS One (2011) 6(3):e18454. 10.1371/journal.pone.0018454 21483788 PMC3069091

[17] Bradshaw A Wickremesekera A Tan ST Peng L Davis PF Itinteang T. Cancer stem cell hierarchy in glioblastoma multiforme. Front Surg (2016) 3:21. 10.3389/fsurg.2016.00021 27148537 PMC4831983

[18] Boyer LA Lee TI Cole MF Johnstone SE Levine SS Zucker JP Core transcriptional regulatory circuitry in human embryonic stem cells. Cell (2005) 122(6):947–56. 10.1016/j.cell.2005.08.020 16153702 PMC3006442

[19] Guo Y Liu S Wang P Zhao S Wang F Bing L Expression profile of embryonic stem cell-associated genes Oct4, Sox2 and Nanog in human gliomas. Histopathology (2011) 59(4):763–75. 10.1111/j.1365-2559.2011.03993.x 22014056

[20] Zhang J Tam W-L Tong GQ Wu Q Chan H-Y Soh B-S Sall4 modulates embryonic stem cell pluripotency and early embryonic development by the transcriptional regulation of Pou5f1. Nat Cell Biol (2006) 8(10):1114–23. 10.1038/ncb1481 16980957

[21] Lim CY Tam W-L Zhang J Ang HS Jia H Lipovich L Sall4 regulates distinct transcription circuitries in different blastocyst-derived stem cell lineages. Cell Stem Cell (2008) 3(5):543–54. 10.1016/j.stem.2008.08.004 18804426

[22] Yang J Chai L Fowles TC Alipio Z Xu D Fink LM Genome-wide analysis reveals Sall4 to be a major regulator of pluripotency in murine-embryonic stem cells. Proc Nat Acad Sci U S A (2008) 105(50):19756–61. 10.1073/pnas.0809321105 19060217 PMC2604985

[23] Wu Q Chen X Zhang J Loh Y-H Low T-Y Zhang W Sall4 interacts with Nanog and co-occupies Nanog genomic sites in embryonic stem cells. J Biol Chem (2006) 281(34):24090–4. 10.1074/jbc.C600122200 16840789

[24] Inghirami G Chiarle R Simmons WJ Piva R Schlessinger K Levy DE New and old functions of STAT3: A Pivitol target for individualized treatment of cancer. Cell Cycle (2005) 4(9):1131–3. 10.4161/cc.4.9.1985 16082218

[25] Niwa H Burdon T Chambers I Smith A. Self-renewal of pluripotent embryonic stem cells is mediated via activation of STAT3. Genes Dev (1998) 12(13):2048–60. 10.1101/gad.12.13.2048 9649508 PMC316954

[26] Tan EM Chudakova DA Davis PF Brasch HD Itinteang T Tan ST. Characterisation of subpopulations of myeloid cells in infantile haemangioma. J Clin Pathol (2015) 68(7):571–4. 10.1136/jclinpath-2014-202846 25834091

[27] Jung C Foerch C Schänzer A Heck A Plate K Seifert V Serum GFAP is a diagnostic marker for glioblastoma multiforme. Brain (2007) 130(12):3336–41. 10.1093/brain/awm263 17998256

[28] Kriegstein A Alvarez-Buylla A. The glial nature of embryonic and adult neural stem cells. Annu Rev Neurosci (2009) 32:149–84. 10.1146/annurev.neuro.051508.135600 19555289 PMC3086722

[29] Pallini R Ricci-Vitiani L Banna GL Signore M Lombardi D Todaro M Cancer stem cell analysis and clinical outcome in patients with glioblastoma multiforme. Clin Cancer Res (2008) 14(24):8205–12. 10.1158/1078-0432.CCR-08-0644 19088037

[30] Zhang L Yan Y Jiang Y Cui Y Zou Y Qian J The expression of SALL4 in patients with gliomas: high level of SALL4 expression is correlated with poor outcome. J Neurooncol (2015) 121(2):261–8. 10.1007/s11060-014-1646-4 25359397

[31] Zeppernick F Ahmadi R Campos B Dictus C Helmke BM Becker N Stem cell marker CD133 affects clinical outcome in glioma patients. Clin Cancer Res (2008) 14(1):123–9. 10.1158/1078-0432.CCR-07-0932 18172261

[32] Vescovi AL Galli R Reynolds BA. Brain tumour stem cells. Nat Rev Cancer (2006) 6(6):425–36. 10.1038/nrc1889 16723989

[33] Annovazzi L Mellai M Caldera V Valente G Schiffer D. SOX2 expression and amplification in gliomas and glioma cell lines. Cancer Genomics Proteomics (2011) 8(3):139–47. 21518820

[34] Mizoguchi M Betensky RA Batchelor TT Bernay DC Louis DN Nutt CL. Activation of STAT3, MAPK, and AKT in malignant astrocytic gliomas: correlation with EGFR status, tumor grade, and survival. J Neuropathol Exp Neurol (2006) 65(12):1181–8. 10.1097/01.jnen.0000248549.14962.b2 17146292

[35] Stieber D Golebiewska A Evers L Lenkiewicz E Brons NH Nicot N Glioblastomas are composed of genetically divergent clones with distinct tumourigenic potential and variable stem cell-associated phenotypes. Acta Neuropathol (2014) 127(2):203–19. 10.1007/s00401-013-1196-4 24154962 PMC3895194

[36] Morokoff A Ng W Gogos A Kaye A. Molecular subtypes, stem cells and heterogeneity: implications for personalised therapy in glioma. J Clin Neurosci (2015) 22(8):1219–26. 10.1016/j.jocn.2015.02.008 25957782

[37] Das S Jena S Levasseur DN. Alternative splicing produces Nanog protein variants with different capacities for self-renewal and pluripotency in embryonic stem cells. J Biol Chem (2011) 286(49):42690–703. 10.1074/jbc.M111.290189 21969378 PMC3234911

[38] Xie X Piao L Cavey GS Old M Teknos TN Mapp AK Phosphorylation of Nanog is essential to regulate Bmi1 and promote tumorigenesis. Oncogene (2014) 33(16):2040–52. 10.1038/onc.2013.173 23708658 PMC3912208

[39] Cai N Li M Qu J Liu GH Izpisua Belmonte JC. Post-translational modulation of pluripotency. J Mol Cell Biol (2012) 4(4):262–5. 10.1093/jmcb/mjs031 22679102

[40] Van Hoof D Munoz J Braam SR Pinkse MW Linding R Heck AJ Phosphorylation dynamics during early differentiation of human embryonic stem cells. Cell Stem Cell (2009) 5(2):214–26. 10.1016/j.stem.2009.05.021 19664995

[41] Jang H Kim TW Yoon S Choi SY Kang TW Kim SY O-GlcNAc regulates pluripotency and reprogramming by directly acting on core components of the pluripotency network. Cell Stem Cell (2012) 11(1):62–74. 10.1016/j.stem.2012.03.001 22608532

[42] Schiffer D Annovazzi L Cassoni P Valentini MC Mazzucco M Mellai M Glioblastoma stem cells: Conversion or reprogramming from tumor non-stem cells? J Stem Cell Res Ther (2015) 5:315 10.4172/2157-7633.1000315

