# Supplementary figures and images for: Cancer Stem Cells in Glioblastoma Multiforme
Source: Front Surg. 2016 Aug 26;3:48. doi: 10.3389/fsurg.2016.00048 (PMC5001191; doi:10.3389/fsurg.2016.00048)

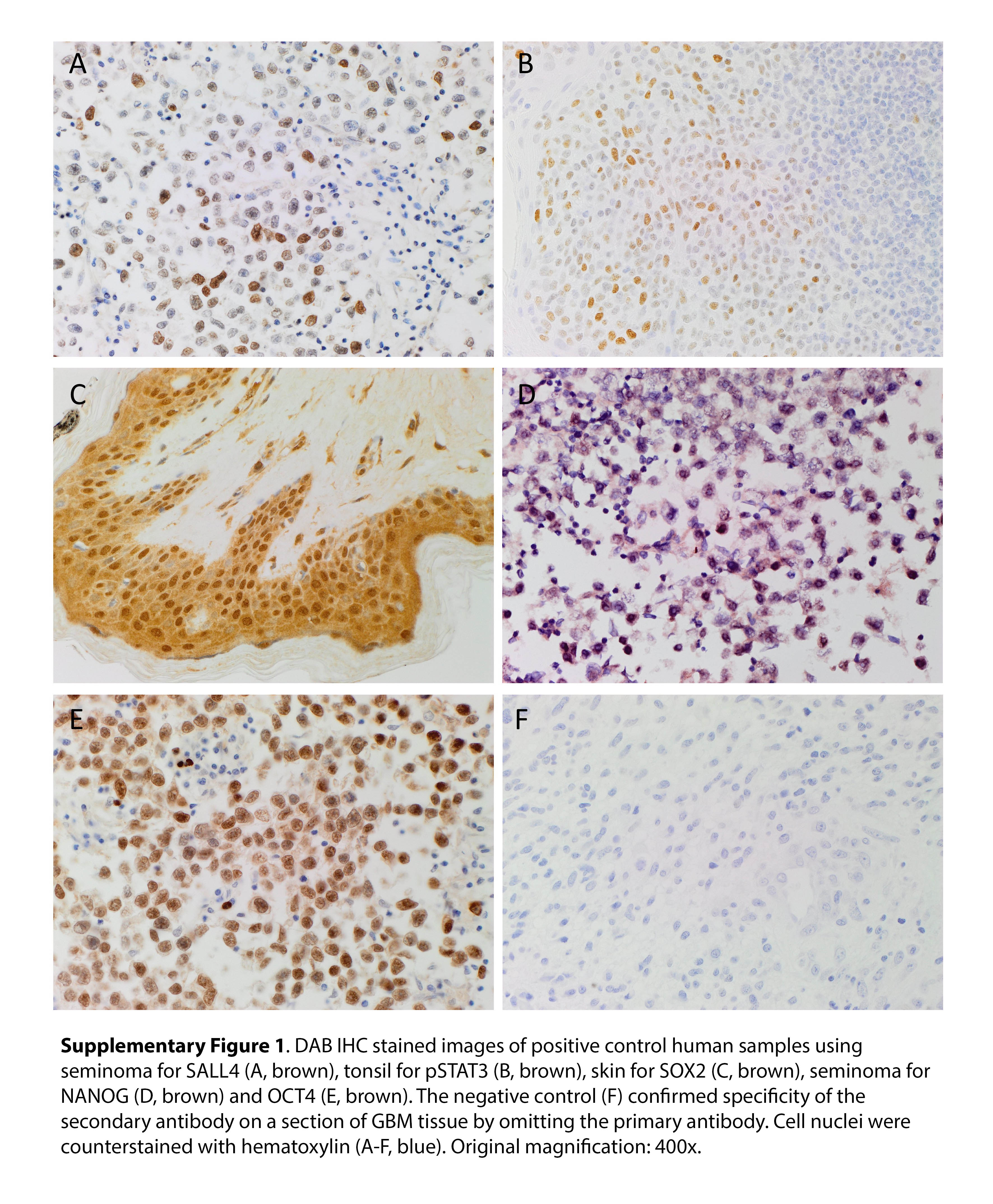

Supplement: Supplementary file 1 [file Image_1.JPEG]

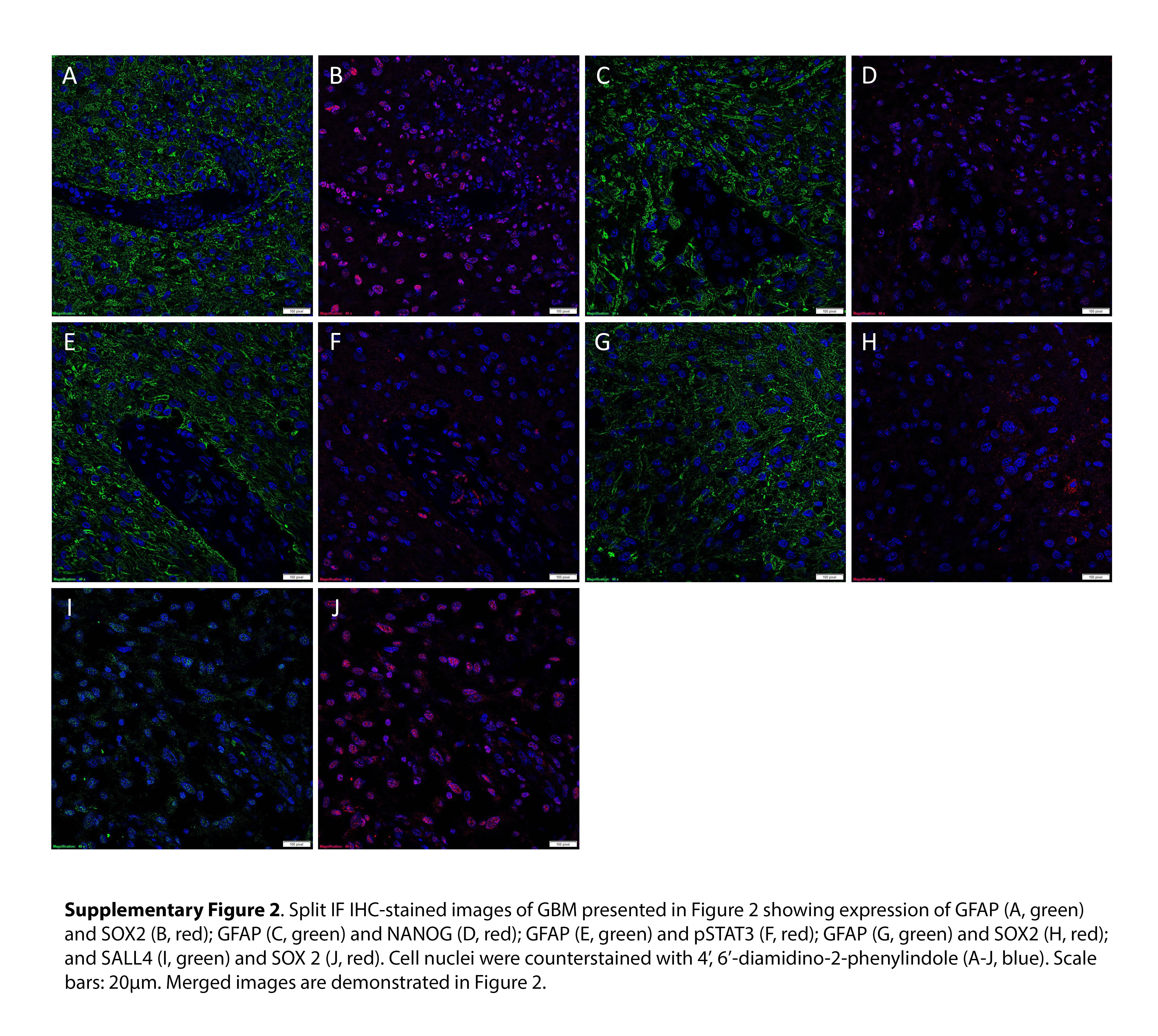

Supplement: Supplementary file 2 [file Image_2.JPEG]
